# Activation of Transcription Factor Nrf2 Signalling by the Sphingosine Kinase Inhibitor SKI-II Is Mediated by the Formation of Keap1 Dimers

**DOI:** 10.1371/journal.pone.0088168

**Published:** 2014-02-05

**Authors:** Nicolas Mercado, Yasuo Kizawa, Keitaro Ueda, Yeping Xiong, Genki Kimura, Audric Moses, Jonathan M. Curtis, Kazuhiro Ito, Peter J. Barnes

**Affiliations:** 1 Airway Disease Section, National Heart and Lung Institute, Imperial College, London, United Kingdom; 2 Department of Physiology and Anatomy, Nihon University School of Pharmacy, Funabashi, Chiba, Japan; 3 Department of Agricultural, Food and Nutritional Sciences, University of Alberta, Edmonton, Alberta, Canada; Christian-Albrechts-University Kiel, Germany

## Abstract

**Background:**

Anti-oxidant capacity is crucial defence against environmental or endogenous oxidative stress. Nuclear factor erythroid 2-related factor 2 (Nrf2) is a redox-sensitive transcription factor that plays a key defensive role against oxidative and cytotoxic stress and cellular senescence. However, Nrf2 signalling is impaired in several aging-related diseases, such as chronic pulmonary obstructive disease (COPD), cancer, and neurodegenerative diseases. Thus, novel therapeutics that enhance Nrf2 signalling are an attractive approach to treat these diseases.

**Methodology/Principal Findings:**

Nrf2 was stabilized by SKI-II (2-(p-hydroxyanilino)-4-(p-chlorophenyl) thiazole), which is a known sphingosine kinase inhibitor, in human bronchial epithelial cell line, BEAS2B, and in primary human bronchial epithelial cells, leading to enhancement of anti-oxidant proteins, such as HO-1, NQO1 and GCLM. The activation of Nrf2 was achieved by the generation of inactive dimerized form of Keap1, a negative regulator of Nrf2 expression, which was independent of sphingosine kinase inhibition. Using mice that were exposed to cigarette smoke, SKI-II induced Nrf2 expression together with HO-1 in their lungs. In addition, SKI-II reduced cigarette smoke mediated oxidative stress, macrophages and neutrophil infiltration and markers of inflammation in mice.

**Conclusions/Significance:**

SKI-II appears to be a novel activator of Nrf2 signalling via the inactivation of Keap1.

## Introduction

Nuclear factor erythroid 2-related factor 2 (Nrf2) is a well characterized redox-sensitive transcription factor that plays a critical defensive role against oxidative and cytotoxic stress [1]. However, defective anti-oxidant and cytoprotective responses due to impaired Nrf2 function have been linked to many important diseases including cancer, aging-related and neurodegenerative diseases, as well as cardiovascular and pulmonary diseases [2]–[8]. For example, chronic obstructive pulmonary disease (COPD), whose major cause is cigarette smoking, shows reduced anti-oxidant capacity and increased oxidative stress that causes glucocorticoid-insensitive airway inflammation [2]. Thus, understanding the molecular mechanism of defective Nrf2 function is critical to the development of novel therapies for several important diseases that are currently poorly treated.

In unstressed cells, Nrf2 is sequestered in the cytoplasm by Keap1 promoting its rapid proteasomal degradation. Nrf2 activation is mediated by electrophiles that target and inhibit cysteine-rich-Keap1, thus inducing Nrf2 accumulation in the nucleus resulting in the activation of multiple antioxidant and cytoprotective genes [9]. Modification of cysteine residues in Keap1 by electrophiles inhibits Keap1 function and stabilizes Nrf2 protein by dissociation from cullin 3 (Cul3), a subunit of the E3 ligase complex-mediated degradation [9], [10]. Nrf2 thus accumulates and translocates to the nucleus where it binds to Antioxidant Response Elements (ARE) as a heterodimer with other members of the basic leucine zipper protein family, such as Maf and Jun [11]. Persistent overload of reactive oxygen species (ROS), such as from cigarette smoke exposure of the lungs, results in chronic inflammation which may lead to COPD [2], which is associated with decreased Nrf2 activity in the lungs [12]. Recently, whole lung tissue and alveolar macrophages from emphysema patients were reported to show decreased Nrf2 protein expression and activity and anti-oxidant genes due to an increase in the negative regulators Keap1 and Bach1 [13]. However, as antioxidant trials have largely failed to provide protection in humans research focus has shifted to activating endogenous antioxidant defences such as Nrf2 [14]. A variety of electrophilic compounds, such as sulforaphane and CDDO-Imidazolide, can activate Nrf2 but they are poorly selective and have toxicity problems, so there is now substantial investment in finding more effective activators [14].

Sphingolipids contribute to various signaling events that can influence cell behavior and function. Sphingolipid metabolites including ceramide, sphingosine, and sphingosine-1-phosphate (S1P) regulate various cellular functions such as survival, inflammation and immunity. [15]. The balance of these metabolites is regulated by members of the sphingosine kinase (SK) family and these are linked to several physiological and pathophysiological processes, including inflammation, aging and cancer [16], [17]. SKs, which includes the two subtypes, SK1 and SK2, can play dynamic roles in the responses of cells to stress such as ROS, leading to modulation of cell fate through a variety of signalling pathways affecting various cellular processes [18]. Several inhibitors of SK have been synthesised. SKI-II ((2-(p-hydroxyanilino)-4-(p-chlorophenyl) thiazole), DMS (N,N-dimethylsphingosine), DHS (d,l,-threo-dihydrosphingosine) are inhibitors of SK1 and SK2 whereas SK1-I (2R,3S,4E)-N**-**methyl-5-(4′-pentylphenyl)-2-aminopent-4-ene-1,3-diol and FTY720 are known inhibitors of SK1 [19]. Recent evidence showed that overexpression of SK1 induces oxidative stress in the heart [20] although the exact role of SK and oxidative stress remains controversial [18]. We hypothesized that inhibition of SK plays a protective role against oxidative stress via activation of Nrf2. SKI-II was shown to be a novel and alternative activator of Nrf2, independently of sphingosine kinase inhibition, with potential benefits for diseases where Keap1 activity is increased such as COPD.

## Results

### SKI-II increased Nrf2 accumulation and anti-oxidant activity

**Figure 1:**
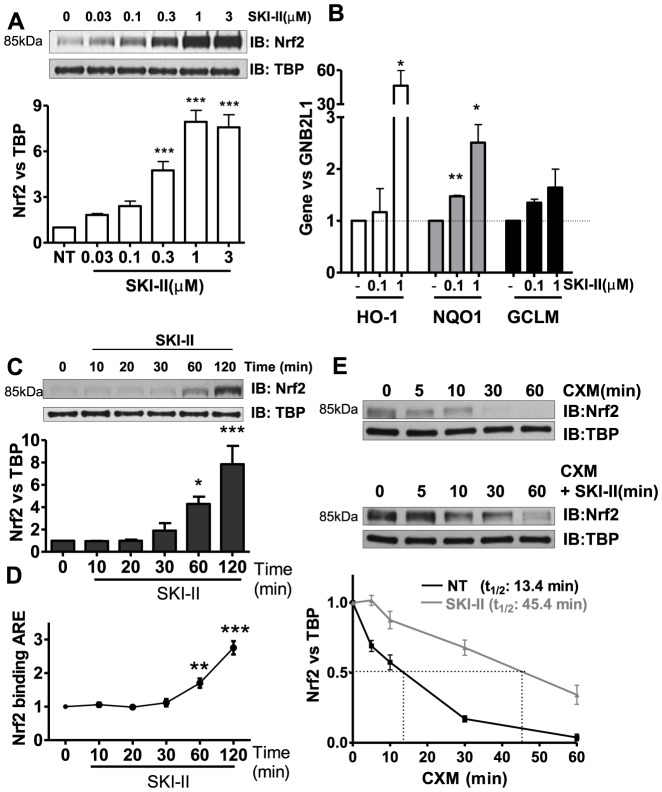
Effect of SK inhibition on Nrf2 in human airway epithelial cells (BEAS2B). **A.** Nuclear extracts from cells treated with increasing concentrations of SK inhibitor SKI-II (0.03 to 3 µM) for 2 h were analysed by immunoblotting for Nrf2 expression and normalized using TBP (fold change vs. NT). *** p<0.0001. **B.** Expression of antioxidant genes NQO1 and GCLM was determined 24 h after and HO-1 after 8 h treatment with SKI-II. GNB2L1 was used as housekeeping gene. ** p<0.001, * p<0.05. **C.** Nuclear fractions from cells treated with SKI-II (1 µM) at increasing times (10–120 min) were analysed for Nrf2 expression and normalized using TBP (nuclear). *** p<0.0001,* p<0.05. **D.** Nuclear fractions from cells treated with SKI-II (1 µM) at increasing times (10–120 min) were analysed for Anti-oxidant Response Element (ARE) binding. *** p<0.0001, ** p<0.001. **E.** BEAS2B cells were treated with cycloheximide (CXM) and SKI-II (1 µM) at different time points (5 to 60 min) and nuclear extracts were analysed for Nrf2 expression normalized against TBP. Data are representative of 3 independent experiments and are means and S.E. of triplicates.

Two hour treatment of SKI-II, concentration-dependently induced Nrf2 protein in nuclei in a bronchial epithelial cell-line (BEAS2B) and it reached almost 9-fold over baseline at 1 µM (Figure 1A). Interestingly, DHS, DMS, SK1-I and FTY270 did not increase Nrf2 expression (Figure S1A). SKI-II did not induce any significant loss in cell viability at concentrations up to 1 µM (Figure S1B). At the same time, SKI-II treatment induced NAD(P)H:quinone oxidoreductase 1 (NQO1), glutamate-cysteine ligase modifier (GCLM) and heme oxygenase-1 (HO-1) by 2.5-, 1.6- and 46-fold, respectively (Figure 1B). Accumulation of Nrf2 protein in the nuclei was seen 30 min after treatment with SKI-II (Figure 1C) and Nrf2 binding to ARE was also detected 60 min after treatment (Figure 1D). Accumulation of Nrf2 protein by SKI-II was associated with prolonged Nrf2 protein half-life by over 3 fold (Figure 1E). Interestingly Sphingosine kinase 1 (SK1) half-life was decreased by SKI-II (Figure S1C).

### SKI-II controls Nrf2 expression

**Figure 2:**
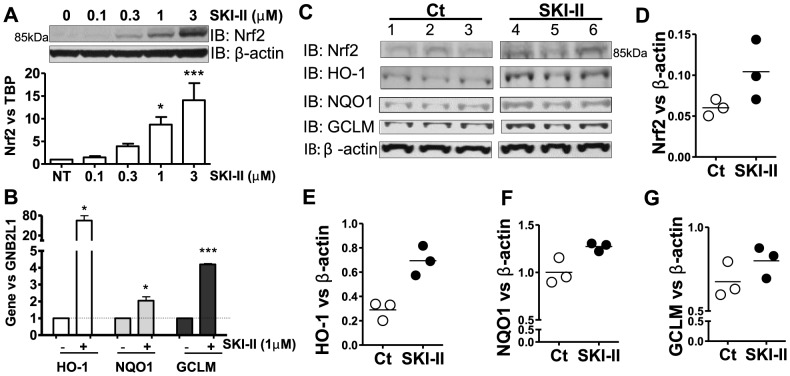
Effect of SKI-II on Nrf2 in primary human airway epithelial cells and in mice *in vivo*. **A.** Whole-cell extracts from normal human bronchial epithelial cells (HBEC) treated with increasing concentrations of SKI-II (0.1 to 3 µM) for 2 h were analysed by immunoblotting for Nrf2 and normalized using β-actin. ** p<0.001, * p<0.05. **B.** Gene expression of NQO1, GCLM and HO-1 were determined after 24 h treatment with SKI-II (1 µM) in HBEC. GNB2L1 was used as housekeeping gene. *** p<0.0001, * p<0.05. **C, D, E, F** and **G.** A/J mice were exposed to nebulized SKI-II (10 µM in PBS, n = 3) or vehicle (Veh, n = 3) for 2 h followed by a further 2 h exposure. Whole-cell extracts were analysed by immunoblotting for Nrf2, GCLM, HO-1 and NQO1 and normalized using β-actin expression. Results are representative of 3 independent experiments and are means and S.E. of triplicates.

We demonstrated that Nrf2 protein accumulation is also induced by SKI-II in primary human bronchial epithelial cells (HBEC) (Figure 2A). Moreover, SKI-II treatment significantly enhanced gene expression of NQO1 (2-fold), HO-1 (>60-fold) and GCLM (>4-fold) (Figure 2B). *In vivo* evidence was obtained from mice that were given intranasal SKI-II for 4 h. As shown in Figure 2C, lung homogenates showed elevated levels of Nrf2 protein together with HO-1, NQO1 and GCLM (Figures 2D, E, F, G).

### SKI-II activation of Nrf2 is not associated with the inhibition of sphingosine kinases

**Figure 3:**
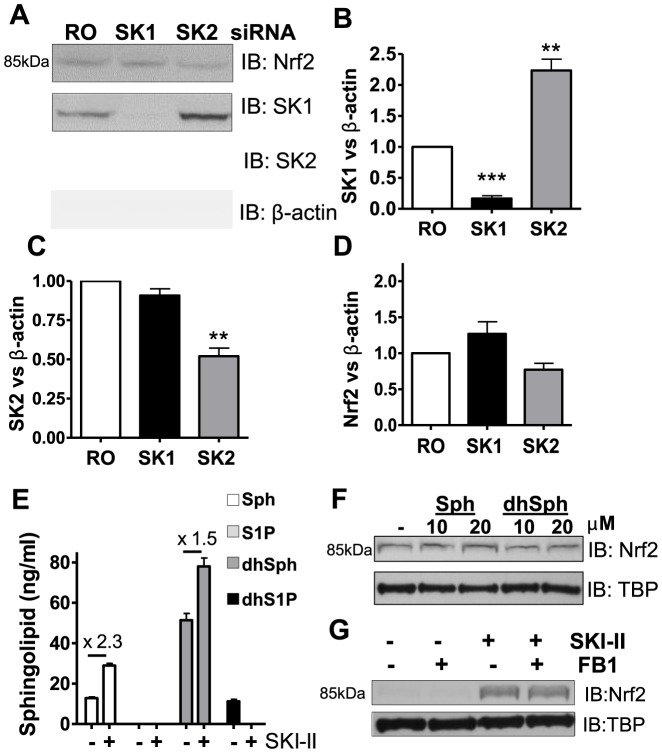
Effect of knocking-down SK1 and SK2 on Nrf2 expression in BEAS2B cells. **A, B, C and D.** Cells transfected with random oligonucleotide (RO) control, SK1 or SK2 siRNA were analysed by immunoblotting (IB) for Nrf2, SK1, SK2, and normalized using β-actin (fold change vs. NT). *** p<0.0001, ** p<0.001, * p<0.05. **E.** BEAS2B cells were stimulated with SKI-II (1 µM) for 2 h, pellets were spiked with C17 sphingosine, dihydrosphyngosine, S1P and dihydroS1P and extracted sphingolipids (C18) determined by LC-MS/MS. Intensity peaks for Sphingosine (Sph), dihydrosphingosine (dhSph) and dyhydrosphingosine 1 phosphate (dhS1P) are indicated in the graph whereas sphingosine 1 phophate levels (S1P) were below the detection range. **F.** Nuclear extracts from cells treated with either sphingosine (10 and 20 µM) or dihydrosphingosine (10 and 20 µM) for 2 h were analysed by immunoblotting for Nrf2 expression and normalized using TBP (fold change vs. NT). **G.** Nuclear extracts from BEAS2B cells treated with fumonisin B1 (FB1; 2 µM) for 1 hour and SKI-II (0.5 µM) for 2 h were analysed by immunoblotting for Nrf2 and TBP expression. Results are representative of two or more independent experiments and are means and S.E. of triplicates.

As SKI-II is not selective for either SK1 or SK2 isoforms, each enzyme isoform was knocked-down (KD) using RNA interference in BEAS2B cells. SK1 was successfully knocked-down by 83% and this did not affect SK2 protein level (Figures 3A, B, C). SK2 was knocked down by 48% (Figures 3A, C) but resulted in SK1 protein expression increase by 2.2-fold compared with random oligonucleotide (RO) (Figures 3A, B, C). As shown in Figures 3A and 3D, SK1 KD did not modify Nrf2 expression as compared to RO control. Similarly, SK2 KD did not result in changes in Nrf2 protein expression. In addition, a double KD of SK1 and SK2 did not result in any modulation of Nrf2 protein levels (Figure S1D). The main lipid substrates for SK1 and SK2 are sphingosine and dihydrosphingosine, which are phosphorylated into S1P and dihydroS1P. The concentration of sphingosine, dihydroshpingosine, S1P and dihydroS1P levels after SKI-II treatment were determined using liquid chromatography and mass spectrometry. As shown in Figures 3E and Figure S1E, dihydrosphingosine (51 ng/ml), sphingosine (12 ng/ml) and dihydroS1P (10 ng/ml) were determined although the concentration of S1P was below the detection limit in non-treated cells (NT). SKI-II treatment augmented the level of dihydrosphingosine by 1.5-fold and sphingosine by 2.3-fold, whereas both S1P and dihydroS1P concentrations were below the limit of detection suggesting inhibition of sphingosine kinase activity. As expected from the KD studies, incubation of BEAS2B cells with sphingosine or dihydropshingosine did not alter Nrf2 expression (Figure 3F). Knock-down of SK1 is reported to increase ceramide levels by the action of ceramide synthase on sphingosine [21], hence we inhibited ceramide synthesis using fumonisin B1 (FB1), a potent inhibitor of ceramide synthase which does not distinguish *de novo* synthesis from the recycling of sphingosine to ceramide [21]. Pre-incubation with FB1 prior to SKI-II treatment did not prevent the increase in Nrf2 protein (Figure 3G), indicating that ceramide is unlikely to be involved in the mechanism of SKI-II-induced Nrf2 protein accumulation. Overall this data suggests that SKI-II stimulation of Nrf2 is independent of the inhibition of sphingosine kinases. In order to address if the effects of SKI-II on cell viability were due to the inhibition of SK1 and SK2 we performed a double SK1 and SK2 KD and looked at cell viability using MTT. We found that although both SK1/2 KDs decreased cell viability over controls (RO), this was not significant (16%) (Figure S1F). Increased concentration of SKI-II which resulted in decreased viability in controls (RO) did not further affect SK1/2 double KD cell viability except at 10 µM SKI-II where cell viability was prevented as compared to controls (Figure S1F). The reason for this remains unknown. In order to verify if SKI-II activation of Nrf2 was responsible for SKI-II concentration dependent reduction in cell viability, we performed an Nrf2 KD (Figure S1F) in BEAS2B cells and treated with increasing concentration of SKI-II. We found that Nrf2 KD significantly reduced baseline cell viability (34%) which stayed more or less constant as compared to control when treated with increasing amounts of SKI-II (Figure S1F). In order to assess if the increase in HO-1 by SKI-II was independent of SK inhibition and dependent on Nrf2, we used the double SK1/2 KD and Nrf2 KD to measure HO-1's ability to be induced by SKI-II. We found that under double SK1/2 KD, SKI-II (1 µM, 8 h) was still able to strongly induce HO-1 (about 24.6 fold) as compared to RO control (25.6 fold). HO-1 activation was however significantly prevented by Nrf2 KD (3.9 fold) (Figure S1G).

### Keap1 inhibition and dimerization by SKI-II is the mechanism of Nrf2 activation

**Figure 4:**
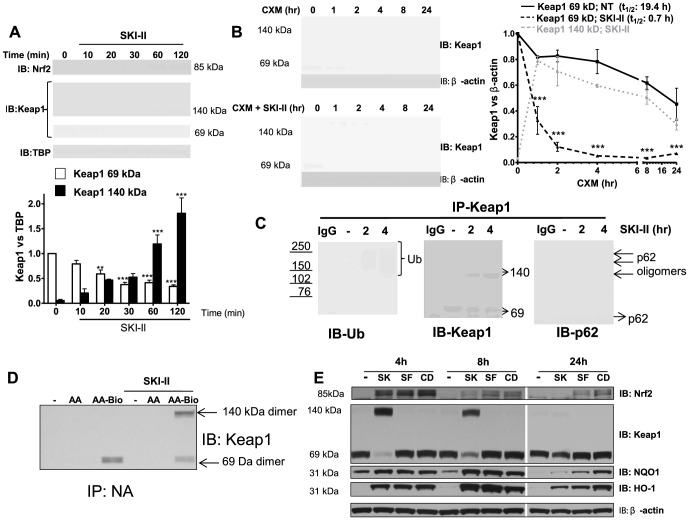
Effect of SKI-II on Keap1. **A.** Nuclear fractions from BEAS2B cells treated with SKI-II (1 µM) at increasing times (10–120 min) were analysed for Keap1 and Nrf2 expression and normalized using TBP (nuclear). Keap1 bands at 140 kDa and 69 kDa were analysed as fold change over non-treatment of the 69 kDa band. *** p<0.0001, ** p<0.001, * p<0.05. **B.** Cells were treated with cycloheximide (CXM) and SKI-II (1 µM) at different time points (1 to 24 h) and whole cell extracts were analysed for Keap1 expression normalized against β-actin. Keap1 bands at 140 kDa and 69 kDa were analysed as fold change over non-treatment of the 69 kDa band. *** p<0.0001 when CXM vs. CXM with SKI-II were compared. **C.** Whole-cell extracts from BEAS2B cells that were treated with SKI-II (1 µM) for 2 and 4 h were immunoprecipitated (IP) using a Keap1 antibody. IP Keap1 was analysed by immunoblotting for ubiquitin modification, p62 and Keap1 expression. IgG: Mouse immunoglobulin control (no cell lysates). Ub: ubiquitin. Hmw: High molecular weight. **D.** Whole-cell extracts from BEAS2B cells that were pre-treated with arachidonic acid (AA; 10 µM) or biotynilated arachidonic acid (AA-Bio; 10 µM) for 30 min were treated with SKI-II (1 µM) for 2.5 h and immunoprecipitated using Neutravidin Agarose Resin (IP-NA). IP-NA was analysed by immunoblotting for Keap1 expression. **E.** Whole-cell extracts from BEAS2B cells treated with SKI-II (SK; 1 µM), sulforaphane (SF; 5 µM) or CDDO-Imidazolide (CD; 50 nM) for 4 h, 8 h and 24 h were analysed by immunoblotting for Nrf2, Keap1, NQO1, HO-1 and β-actin. Results are representative of 3 independent experiments and are means and S.E. of triplicates.

There was no evidence of initial Nrf2 build-up in the cytoplasm at least 10 min after SKI-II treatment, suggesting that Nrf2 nuclear translocation occurred immediately after activation as shown before [22] (Figure S2A). In parallel, Keap1, which was found both in the cytoplasm and nucleus, showed progressive loss of the monomeric (69 kDa) active form of Keap1 with formation of dimerized Keap1 (140 kDa) that was resistant to the reducing conditions of SDS-PAGE (Figure 4A). Thus, the molecular mechanisms of SKI-II on Nrf2 accumulation appears to be via induction of inactive dimerized Keap1. Although the formation of Keap1 dimer is an irreversible process that involves covalent bonding, Nrf2 protein stimulation induced by SKI-II peaked after 2 h and gradually returned to basal levels by 24 h (Figure S2B). At same time, 140 kDa bands of Keap1 gradually disappeared as the monomeric form was restored (Figure S2B). When BEAS2B cells were treated with SKI-II in the presence of cyclohexamine, we confirmed that Keap1 half-life (t_1/2_) was decreased from 19.4 h to only 0.7 h (Figure 4B). Dimerized Keap1 was also reduced over time suggesting continuous degradation of the dimerized form (Figure 4B). In fact, immunoprecipitated Keap1 from cells treated with SKI-II for 2 and 4 h, showed an increase in dimerized Keap1 with concomitant reduction of the monomeric form (Figure 4C). The increase in dimerized Keap1 was associated with increase in ubiquitinated bands of Keap1. Additionally, p62, which mediates Keap1 degradation by autophagy, and which oligomerizes when activated [23], [24], was associated with Keap1 after SKI-II treatment (Figure 4C). As SKI-II has been shown to decrease androgen expression via the activation of oxidative stress [25] we measured the effect of SKI-II on intracellular ROS production using DCF. SKI-II treatment for 2 h did not induce ROS production whereas hydrogen peroxide stimulated ROS in a dose dependent manner (Figure S2C). In addition, total antioxidant capacity (TAC) by SKI-II was increased (18%) in BEAS2B cells as measured by reduction of copper (II) to copper (I). Similarly, N-acetyl cysteine pre-treatment of BEAS2Bs also induced an increase of 29% in TAC activity whereas cigarette smoke treatment, at a concentration in which cell viability was not affected (CS; 3.5% v/v) reduced TAC activity by 22% (Figures S2D and S2E). In addition, we found that pre-incubation with glutathione (GSH) prevented Nrf2 activation via inhibition of Keap1 dimers (Figure S2F). This suggested that SKI-II induced unknown endogenous electrophilic compounds that could bind cysteine residues in Keap1 and that excess of GSH could compete against Keap1. The arachidonic acid (AA) pathway is a source of endogenous electrophilic fatty acids capable of activating Nrf2 [26]. Hence in order to verify if an AA derivative can bind Keap1, biotynilated AA was incubated with BEAS2B cells and results suggested an incorporation of processed biotin-AA derivative into Keap1 monomers. Interestingly, addition of SKI-II further enhanced incorporation of processed biotin-AA derivative into Keap1 dimers (Figure 4D). This was not observed with the non-biotynlated AA (Figure 4D). This data suggests that an unknown electrophile derivative from AA, induced by SKI-II could activate Nrf2 via Keap1 inhibition and dimer formation.

In order to compare the effects of SKI-II with other Nrf2-activators, we investigated the effect of sulforaphane (SF) and CDDO-Immidazole (CDDO-Im) on BEAS2B cells. We used the maximum concentrations of SF (5 µM) and CDDO-Im (50 nM) that did not affect cell viability and compared it with SKI-II (1 µM) at increasing time points (4, 8 and 24 h) (Figures S2G, S2H). CDDO-Im prolonged Nrf2 stability and subsequent induction of HO-1 and NQO1 the longest as compared to SF and SKI-II, however, these last two showed a bigger peak of activation for HO-1 and NQO1 at 8 hours (Figure 4E). Only SKI-II was able to induce Keap1 dimers suggesting a different mechanism of activation (Figure 4E). We propose that SKI-II is a novel activator of Nrf2 that induces Keap1 dimer formation possibly by induction of unknown electrophilic compounds.

### Effects of SKI-II on Nrf2, HO-1 and other inflammatory markers in CS-exposed mice

**Figure 5:**
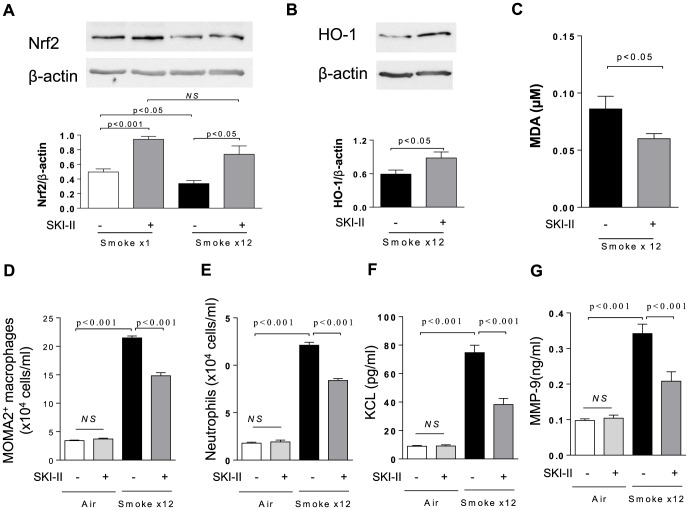
Effects of intranasal SKI-II on Nrf2, HO-1 and other inflammatory markers in cigarette smoke exposed mice. Nrf2 protein level normalized to β-actin in lung. Mice were exposed to smoke only once or 12 times (once daily). The lung was collected 2 h after cigarette smoke exposure. **B.** HO-1 protein in lung. The Western Blot image from pooled samples was shown. **C.** MDA in BALF, **D.** Alveolar macrophage in BALF, **E.** Neutrophils in BALF, **F.** CXCL1(KC) in BALF, **G.** MMP9 in BALF. Mice were treated with SKI-II (10 µM), intranasally before each cigarette smoke. Results are representative of 4–5 independent experiments and are means and S.E. of triplicates.

As shown in Figure 5A, the level of Nrf2 normalized to β-actin was significantly lower in the lung from mice exposed to CS for 12 days than that from mice exposed to CS only once. Intranasal SKI-II treatment significantly increased Nrf2 level in both conditions. In 12 days-smoke model, HO-1 expression protein in lung was also increased by SKI-II treatment (Figure 5B), and conversely, SKI-II significantly reduced the level of malondialdehyde (MDA), an oxidative stress marker, in BALF (Figure 5C). Interestingly, SKI-II treatment also significantly reduced the number of alveolar macrophage and neutrophils that were induced by 12 days-smoke model (Figure 5D and 5F). These finding correlated with a SKI-II reduction of CS-induced KC and MMP-9, respectively a neutrophil chemo-attractant chemokine and a macrophage/neutrophil secreted protease (Figure 5G).

## Discussion

Nrf2 production appears to decline with age, while free radical production increases [27]. Thus the regulation of Nrf2 may be key to the management of the so-called ‘diseases of aging’, which include cardiovascular disease, neurodegenerative diseases, cancer, type 2 diabetes, chronic failure of the kidneys and heart and COPD [14], [28]. COPD is an example of a disease of accelerated aging where Nrf2 is decreased [28]. Defective Nrf2 can explain the excessive oxidative stress present in the lungs that causes damage and inflammation [28]. In fact, decreased Nrf2 has been shown to be associated with defective HO-1, GCLM and NQO1 in lung biopsies from patients with COPD [12]. We have shown that SKI-II has the potential to restore redox homeostasis by increasing antioxidant response element-mediated (ARE) expression of phase II antioxidant enzymes. Nrf2 protein was stabilized by SKI-II treatment resulting in nuclear build-up and increased binding to ARE. The increase of Nrf2 half-life was associated with a dramatic decrease in Keap1 stability. Moreover, macrophages from COPD patients have shown that Nrf2 expression is reduced due to increased Keap1 expression [13], suggesting that a reduction of Keap1 by SKI-II could restore Nrf2 levels in COPD macrophages. Indeed, deletion of Keap1 attenuates acute cigarette smoke-induced oxidative stress and inflammation in the lung [29]. SKI-II-induced Nrf2 expression and activation of cytoprotective gene expression was further confirmed in primary human bronchial epithelial cells (HBEC) and *in vivo* using intranasal SKI-II treatment in mice. Other studies *in vivo* using rodent models have already shown that dietary Nrf2 activators, such as the isothiocyanate sulforaphane found in broccoli, can increase antioxidant defences, reduce blood pressure and inhibit pro-inflammatory signalling pathways in the kidney [30]. Another Nrf2 activator, CDDO-Imidazolide, attenuated cigarette smoke-induced emphysema and cardiac dysfunction in mice.[31]. However, both sulforaphane and CDDO-Imidazolide are known to have many off-target effects and toxicity [32], [33]. We also showed that in a 12 days-smoke model using mice, Nrf2 was reduced as compare to a day exposure thus mimicking what is observed in COPD [12], [34]. SKI-II was able to restore normal Nrf2 expression as well as reduce oxidative stress generated by CS exposure. Interestingly, the recruitment of neutrophils and macrophages, characteristic in COPD together with increased production of MMP-9 and KC (human CXCL-8 equivalent) were also reduced by SKI-II [2]. The present study suggests that SKI-II is a novel Nrf2 activator that could play a potential therapeutic role in COPD.

Interestingly, other known inhibitors of SK did not activate Nrf2. In addition, specific KD of SK1 and SK2 suggested that the inhibition of both kinases were not important for Nrf2 activation. In fact, SKI-II induced early changes in the balance of sphingolipids resulting in an expected increase in sphingosine and dihydrosphingosine. Exogenous addition of these did not affect Nrf2 stability either. Inhibition of sphingosine kinase has been reported to increase ceramide production via ceramide synthase. Inhibition of this enzyme by fumonisin B1 thus preventing a build-up in ceramide did not affect SKI-II induction of Nrf2 thus ruling out a role for this sphingolipid. SKI-II is a non-ATP competitor inhibitor of SK1 and SK2 that has been previously show to decrease SK1 stability and this was confirmed in our study [35]. FTY720, which is also known to reduce SK1 expression [36], did not activate Nrf2. Our data confirmed a novel role for SKI-II independent of the sphingolipid biostat.

When comparing the effect of SKI-II on Keap1 dimerization to sulforaphane and CDDO-Imidazolide, only SKI-II was able to decrease Keap1 levels while inducing Keap1 dimers. A decrease in Keap1 expression has been previously observed using a polyphenolic activator of Nrf2, quercetin [37]. In parallel to Keap1 reduction, the rapid increase of Keap1 dimer structure was resistant to the reducing conditions of SDS-PAGE. Formation of this structure that is resistant to reducing agents has been reported by electrophilic compounds and the structure was identified as a Keap1 homodimer [38], [39]. The stability of this modified structure under reducing conditions from SDS-PAGE indicated that the dimer form is not simply a disulphide-linked dimeric complex, but involves a stronger covalent bond. Only 10 minutes after SKI-II treatment an increase in Keap1 dimers was observed. The speed of this reaction suggested a possible induction of reactive oxygen species (ROS) that could be targeting Keap1. Recently, Tonelli *et al.* suggested that SKI-II reduced androgen receptor (AR) via activation of ROS [25]. However ROS production was unaffected 2 h after SKI-II treatment and the total anti-oxidant capacity was increased after 24 h suggesting that Nrf2 anti-oxidant genes increased reducing conditions in the cell. The work from Tonelli *et al.* used 10× more SKI-II and pre-incubation of anti-oxidant NAC prevented SKI-II reduction of AR. I also showed that GSH prevented SKI-II induction of Keap1 dimers however the suggestion is that cysteine from GSH compete against the cysteines from Keap1 for the binding to unknown electrophiles as previously shown before [40]. Interestingly, an endogenous source of electrophilic compounds able to induce Keap1 dimers whilst activating Nrf2 is the arachidonic acid pathway [40]. Our study suggests that SKI-II might regulate the arachidonic acid pathway resulting in the generation of electrophilic fatty acids (EFA). Although we have not identified the EFA, it is known that derivatives from enzymatic modulation of arachidonic acids results in the generation of potent anti-inflammatory Nrf2 activators such as 15-oxo-ETE, Lipoxin A4 or 15d-PGJ2 [26], [41], [42]. However, additional unknown EFA might be generated and further studies will be needed in order identify them. In addition, we showed that although SKI-II induced a covalent modification, the dimer degrades slowly by autophagy and does not revert to a monomer structure. The accumulation of ubiquitinated Keap1 aggregates result in recognition and binding by the autophagy adaptor protein p62. The ensuing oligomerization was directed towards lysosomal digestion by autophagy [24]. In fact, previous findings also showed that electrophile-induced Keap1 homodimers are ubiquitinated and degraded [38], [39]. Covalent modifications by EFA could be deleterious in cells however adequate removal of Keap1 dimers by autophagy prevented any toxicity and allowed a sustained and prolonged anti-oxidant signal that could be beneficial in diseases where anti-oxidant defences are compromised.

In summary, SKI-II impairs Keap1 function by induction of dimerized Keap1, leading to its degradation and clearance by autophagy. This results in a prolonged Nrf2 protein accumulation and subsequent activation of cytoprotective and antioxidant genes. SKI-II provides a new mechanism that could lead to a novel approach for managing oxidative stress-mediated diseases such as COPD.

## Materials and Methods

The animal study was approved by the Ethics Review Committee for Animal Experimentation of Nihon University.

### Reagents and antibodies

Sphingosine, SKI-II (2-(p-Hydroxyanilino)-4-(p-chlorophenyl) thiazole), DMS (N,N-Dimethylsphingosine), DHS (dihydro-sphingosine ), FTY720 and SK1-I ((2R,3S,4E)-N**-**methyl-5-(4′-pentylphenyl)-2-aminopent-4-ene-1,3-diol) were obtained from Echelon Biosciences (Salt Lake City, UT). Hydrogen peroxide,3-(4,5-dimethylthiazol-2 yr)-2-5-diphenyl tetrazoliumbromide (MTT)sulforaphane,CDDO-Imidazolide and propidium were from Sigma (Poole, UK). Dihydrospingosine was from Tocris Bioscience (Bristol, UK). S1P, dihydroS1P and fumonisin B1 were from Enzo Life Sciences International (Exeter, UK). C17s sphingosine, dihydrosphyngosine, S1P and dihydroS1P were purchased from Avanti Polar Lipids (Alabaster, AL). FITC-conjugated anti-macrophage (MOMA2) antibody and anti-neutrophils (7/4) antibody were purchased from Acris Antibodies (GmbH, Herford, Germany). Arachidonic acid and Biotin-Arachidonic acid were purchased from Cayman (Ann Arbor, MI). Neutravidin Agarose Resin was purchased from Thermo Scientist (East Riding of Yorkshire, UK). Antibodies against the following were used for immunoblotting: β-actin, p62, NQO1, ubiquitin (Sigma), Nrf2 (Santa Cruz Biotechnology, Santa Cruz, CA), Nrf2, GCLM, HO-1, TBP, β-actin, SK2 (Abcam, Cambridge, UK), SK1 (Abgent, San Diego, CA) and Keap1 (Origene, Rockville, MD).

### Cell culture and transfections

BEAS2B cells (human airway epithelial) (ATCC Teddington, UK) were cultured as previously described [43]. Normal human bronchial epithelial cells (HBEC) were purchased from Lonza (Basel, Switzerland) and cultured as specified by the company. Cells were serum starved 24 h before stimulation. Cells were transfected with HiPerfect for siRNA and SK1, SK2 and Nrf2 were knocked-down with sequence-specific siRNA from Qiagen (Crawley, UK) using All-Stars randon oligonucleotide as control, as previously described [43]. For Nrf2 protein stability experiment, BEAS2B cells were stimulated with cycloheximide (Sigma) (0.5 µg/ml) from 5 to 60 min in the presence of 30 min pre-incubation with SKI-II (0.5 µM). For Keap1 protein stability experiment, BEAS2B cells were stimulated with cycloheximide (Sigma) (0.5 µg/ml) from 1 to 24 h in the presence of SKI-II (1 µM). Cell toxicity was determined by MTT assay.

### Preparation of cigarette smoke extracts

One full-strength Marlboro cigarette with filter removed (Phillip Morris USA, Richmond, VA) was bubbled into 10 mL of serum-free culture media at a rate of one cigarette per 1.5 minutes. Cigarette smoke extract (CSE) was then passed through a 0.2 µm filter to sterilize and remove particulate matter and was used immediately. The optical density was measured at 320λ (OD 0.81±0.07). The concentration of cigarette smoke extract (CSE) used was calculated as % v/v of CSE vs. total volume of media.

### Real-time quantitative PCR

Total cellular RNA was extracted and cDNA was prepared as previously reported [43]. HO-1, GCLM, NQO1 and GNB2L1 analysis was performed using probe sets from Qiagen in the Corbett Rotor Gene 6000.

### Western Blotting and Immunoprecipitation

Protein extracts were prepared using modified RIPA buffer (50 mM Tris HCL pH 7.4, 0.5% NP-40, 0.5% w/v Na-deoxycholate, 150 mM NaCl with complete protease (Roche, Welwyn Garden City, UK). N-ethylmaleimide (25 mM, Sigma) was also added to the RIPA buffer for immunoprecipitation of Keap1 to look at ubiquitin. Nuclear extraction was performed using Active Motif kit (Rixensart, Belgium). Immunoprecipitation was conducted with anti-Keap1 or anti-Nrf2 in 500-1000 µg of cell lysate overnight at 4°C. Immunoprecipitates were captured with mouse TrueBlot IP beads (eBioscience, Hatfield, UK). After extensive washing, bound proteins were released by boiling in SDS–PAGE sample buffer. Protein extracts (40 µg or immunoprecipitates) were analysed by SDS-PAGE (Invitrogen, Paisley, UK) and detected with Western blot analysis by chemiluminescence (ECL Plus; GE Healthcare, Hatfield, UK). Immunoprecipitation of BEAS2Bs treated with Biotin-Arachidonic acid was conducted with Neutravidin Agarose Resin in 750 µg of cell lysate overnight at 4°C.Protein expression levels were expressed relative to β-actin or TBP expression.

### Nrf2 activity assay

Cells were stimulated SKI-II for different time points and nuclear extracts were used for the determination of Nrf2 binding activity to immobilized anti-oxidant response elements (ARE) using a TransAM™ Nrf2 kit (Active Motif).

### Quantification of sphingolipids by mass spectrometry

Cells were treated with SKI-II(1 µM) for 2 h then collected and washed in cold PBS. The pellet was fortified using 50 µl of C17 sphingolipid standards solution containing C17- sphingosine (Sph), dihydrosphingosine (dihydroSph), S1P and dihydroS1P. Sphingolipids were extracted using a mixture of iso-propanol/deionised water/ethyl acetate at 30:10:60 v/v/v. The organic phase was evaporated to dryness using a N_2_ evaporator. Pellets were re-suspended in a solution of 1 mM ammonium formate in methanol containing 0.2% formic acid and transferred to a vial for analysis by HPLC. Sphingolipids were quantified by liquid chromatography combined with electrospray ionization-tandem mass spectrometry (LC-ESI-MS/MS). An Agilent 1200 HPLC was used coupled to an AB-Sciex 3200 QTrap mass spectrometer (AB Sciex, Concord, ON, Canada) employing Analyst v1.4.2 software. A 7.5 cm ×2.1 mm Ascentis Express C18 HPLC column (Supelco, Bellefonte, PA) with a particle size of 2.7 µm was used with a mobile phase A of MeOH with 0.2% formic acid and mobile phase B of 50 mM ammonium formate at pH 3. The gradient conditions were: 0–0.1 min, 75%A; 0.1–8 min 75%A–95%A; 8–10 min 95%A; 10.1–15 min 75%A; flow rate: 250 µl/min (400 µl/min from 10.2 min to 12.5 min). Mass spectrometry in the positive ion mode with multiple reactions monitoring (MRM) was used to quantify sphingolipids using the following transitions at optimized collision energies: C18Sph (*m*/*z* 300→282); C18dhSph (*m*/*z* 302→284); C18S1P (*m*/*z* 380→264); C18dhS1P (*m*/*z* 382→284); C17Sph (*m*/*z* 286→268); C17dhSph (*m*/*z* 288→270); C17S1P (*m*/*z* 366→250). Quantification of sphingolipids was achieved by measuring the peak area ratios of sphingolipid to C17 internal standard and response factors from calibration curves obtained using authentic sphingolipid standards.

### Total anti-oxidant capacity assay

Cells were stimulated SKI-II for 24 hours and 20 µg of protein from whole extracts were used for the determination of the total anti-oxidant capacity using OxiSelect™ Total Antioxidant Capacity (TAC) Assay Kit (Cell Biolabs, San Diego, CA). Cells treated with CSE (3.5%) or N-acetyl cysteine (NAC, 10 mM) were used as controls 30 minutes before collection.

### Intracellular ROS production

Cells were stimulated with SKI-II for 2 hours and incubated in the presence of 10 µM 2′-7′-dichlorofluorescin diacetate (DCF-DA, Cell Biolabs) for 30 minutes using H_2_O_2_ (50–500 µM) as control. Fluorescence was measured using a microplate fluorescence reader at excitation wavelength of 485 nm and emission wavelength of 530 nm.

### Animal study

Specific pathogen-free A/J mice (male, 5 weeks old) were purchased from Sankyo Labo Service Co. Ltd. (Tokyo, Japan) and adapted for 1 week in a temperature (24±1°C) and humidity (55±5%) controlled room with a 12 h day-night cycle. The mice were reared on a standard diet and tap water ad libitum. All animal studies were performed in accordance with the guidelines of the Nihon University Animal Care and Use Committee.

Mice were treated with SKI-II (10 µM in PBS) or vehicle intranasally twice at 2 hourly intervals before lung collection. Each whole right lung was homogenized in 500 µl of RIPA buffer (10 mM Tris-HCl [pH 7.4], 1% Triton X-100, 0.5% sodium deoxycholate, 0.1% SDS, 150 mM NaCl, 1 mM EDTA, 0.5 mM PMSF, 0.5 mM sodium orthovanadate, 10 ug/ml leupeptin, 25 µg/ml aprotinin, 10 µg/ml pepstatin A, 2.5 mM NaF and 0.5 mM sodium pyrophosphate) and 40 µg of cell extracts were analysed by SDS-PAGE.

For the cigarette smoke experiment, A/J mice (4 – 5 mice per group) were exposed cigarette smoke (4% cigarette smoke diluent with compressed air) or fresh air for 30 min/day for 12 days using the commercially marketed filtered Hi-lite cigarettes (17 mg of tar and 1.4 mg of nicotine per cigarette; Japan Tobacco Inc., Tokyo, Japan) and using a Tobacco Smoke Inhalation Experiment System for small animals (Model SIS-CS200; Sibata Scientific Technology, Tokyo, Japan) as described previously [34]. SKI-II was dissolved in 1% DMSO in saline. SKI-II (10 µM) or vehicle was administered intranasally in a volume of 35 µl under anaesthesia with 3% isoflurane at 30 min before each cigarette smoke exposure.

#### Bronchoalveolar lavage fluid

BALF was collected at 2 h after the last cigarette smoke or fresh air exposure as previously reported [34]. The BALF was centrifuged at 500×g for 10 min at 4°C. The cell pellet was resuspended in 0.2% NaCl to induce hemolysis of erythrocytes. After isotonization by adding same volume of 1.6% NaCl, the total number of BAL cells was counted and aliquoted for flow cytometry analysis. The lung was also removed, and stored at −80°C for immunoblotting.

### ELISA

Murine CXCL1 (KC), MMP9 and MDA in BALF supernatant were determined using a Quantikine mouse CXCL1/KC and Quantikine total MMP9 ELISA kit (R&D Systems, Minneapolis, MN) and Oxiselect TBARS assay (MDA quantitation) kit (Cell Biolabs Inc., San Diego, CA).

### Statistical Analysis

Data are expressed as median ± SEM. Results were analysed using t-test and one- or two-way ANOVA for repeated measures with Dunnett or Bonferroni post-tests using the Graph Pad Prism Software (Prism, San Diego, CA) was used for statistical calculations. P<0.05 was considered statistically significant.

## Supporting Information

Figure S1 **A. Effect of SK inhibition on Nrf2 in human airway epithelial cells (BEAS2B).** Nuclear or whole cell extracts from cells treated with increasing concentrations of SK inhibitors DHS (0.3 to 30 µM), DMS (0.1 to 10 µM), SK1-I (0.1 to 10 µM) and FTY720 (0.1 to 10 µM) for 2 h were analysed by immunoblotting for Nrf2 expression and normalized using TBP or β-actin. **B.** BEAS2B cells were analysed for cell viability using an MTT assay 24 hours after SKI-II treatment. *** p<0.0001. **C.** BEAS2B cells were treated with cycloheximide (CXM) and SKI-II (1 µM) at different time points (1 to 24 h) and whole cell extracts were analysed for sphingosine kinase 1 (SK1) expression and β-actin. **D.** Cells transfected with random oligonucleotide (RO) control, SK1, SK2 and SK1+SK2 siRNA were analysed by immunoblotting (IB) for Nrf2, SK1, SK2 and β-actin. **E.** BEAS2B cells were stimulated with SKI-II (1 µM) for 2 h, pellets were spiked with C17 sphingosine, dihydrosphyngosine, S1P and dihydroS1P and extracted sphingolipids (C18) determined by LC-MS/MS. Intensity peaks for C17 and C18 sphingolipids are indicated in the graphs. Chromatograms show MRM traces as described in the Methods section. The higher levels observed for C18Sph and C18dhSph for the SKI-II treatment compared to NT can be seen from these chromatograms. **F.** Cells transfected with random oligonucleotide (RO) control, SK1+SK2 and Nrf2 siRNA were treated 24 h with SKI-II (0.1 to 10 µM) and analysed for cell viability using an MTT assay. Nrf2 KD was verified by immunoblotting (IB) for Nrf2 against β-actin. **G.** Cells transfected with random oligonucleotide (RO) control, SK1+SK2 and Nrf2 siRNA were treated 8 h with SKI-II (1 µM) and analysed for HO-1 expression by qRT-PCR.
(PPTX)

Figure S2 **A. Cytoplasmic fractions from BEAS2B treated with SKI-II (1 µM) at increasing times (10–120 min) were analysed for Keap1 and Nrf2 expression and normalized against β-actin (cytoplasmic).** Keap1 bands at 140 kDa and 69 kDa were analysed as fold change over non-treatment of the 69 kDa band, *** p<0.0001, ** p<0.001, * p<0.05. **B.** Nuclear and cytoplasmic fractions from BEAS2B cells treated with SKI-II (0.5 µM) at increasing times (2–24 h) were analysed for the expressions of Keap1, Nrf2, TBP (nuclear) and β-actin (cytoplasmic). **C.** Whole cell extracts from BEAS2B cells were stimulated with SKI-II for 2 h and incubated in the presence of 10 µM 2′-7′-dichlorofluorescin diacetate (DCF-DA) for 30 min using H_2_O_2_ (50–500 µM) as control. **D.** Whole cell extracts (20 µg) from BEAS2B cells were stimulated SKI-II for 24 h used for the determination of the total anti-oxidant capacity by measuring the reduction of copper (II) to copper (I) against in µM copper reducing equivalents (CRE). Cells were also treated with cigarette smoke extract (3.5% v/v of 1 filtered cigarette into 10 ml of media) or N-acetyl cysteine (NAC, 10 mM) as controls 30 min before collection. **E.** BEAS2B cells were analysed for cell viability using an MTT assay 24 h after CSE treatment (3.5, 7.5 and 9% v/v) **F.** Whole-cell extracts from BEAS2B cells pre-treated with glutathione (GSH; 5 and 10 µM) before treatment with SKI-II (1 µM) for 2 h were analysed for Keap1 and Nrf2 expression and normalized against β-actin. **G.** BEAS2B cells were analysed for cell viability using an MTT assay 24 h after sulforpahane treatment (SF; 1 to 50 µM) or **H.**CDDO-Imidazolide (CDDO-Im; 10-250 nM) *** p<0.0001.
(PPTX)
