# Correction: Activation of Transcription Factor Nrf2 Signalling by the Sphingosine Kinase Inhibitor SKI-II Is Mediated by the Formation of Keap1 Dimers

**DOI:** 10.1371/journal.pone.0097208

**Published:** 2014-05-02

**Authors:** The PLOS ONE Staff

Some of the figures in this article are incorrect. The authors have provided higher resolution images for Figures 1, 2, 3, 4, and 5, and SI Figures 1 and 2. Please view these figures here.

**Figure 1:**
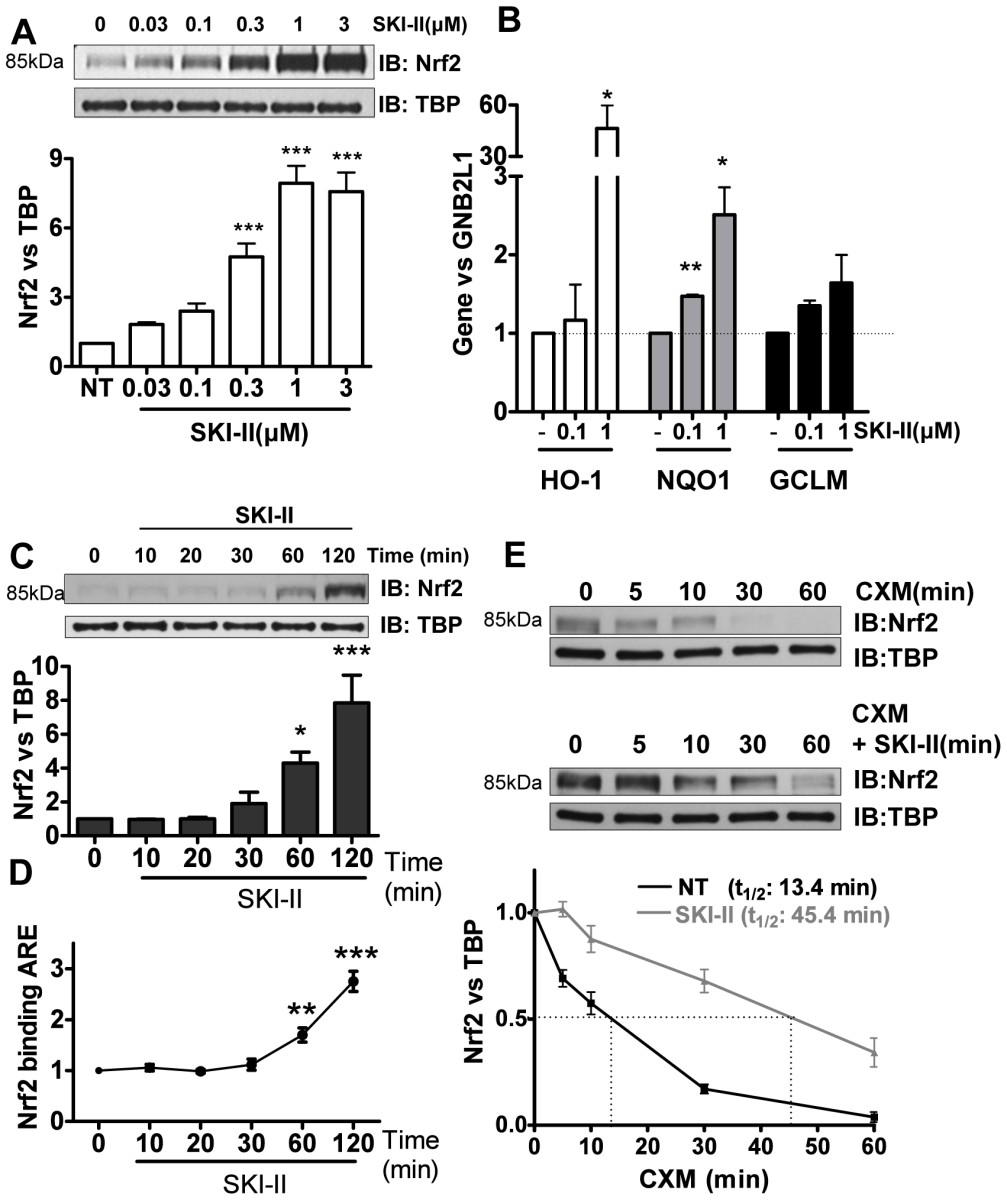
Effect of SK inhibition on Nrf2 in human airway epithelial cells (BEAS2B). **A.** Nuclear extracts from cells treated with increasing concentrations of SK inhibitor SKI-II (0.03 to 3 µM) for 2 h were analysed by immunoblotting for Nrf2 expression and normalized using TBP (fold change vs. NT). *** p<0.0001. **B.** Expression of antioxidant genes NQO1 and GCLM was determined 24 h after and HO-1 after 8 h treatment with SKI-II. GNB2L1 was used as housekeeping gene. ** p<0.001, * p<0.05. **C.** Nuclear fractions from cells treated with SKI-II (1 µM) at increasing times (10–120 min) were analysed for Nrf2 expression and normalized using TBP (nuclear). *** p<0.0001,* p<0.05. **D.** Nuclear fractions from cells treated with SKI-II (1 µM) at increasing times (10–120 min) were analysed for Anti-oxidant Response Element (ARE) binding. *** p<0.0001, ** p<0.001. **E.** BEAS2B cells were treated with cycloheximide (CXM) and SKI-II (1 µM) at different time points (5 to 60 min) and nuclear extracts were analysed for Nrf2 expression normalized against TBP. Data are representative of 3 independent experiments and are means and S.E. of triplicates.

**Figure 2:**
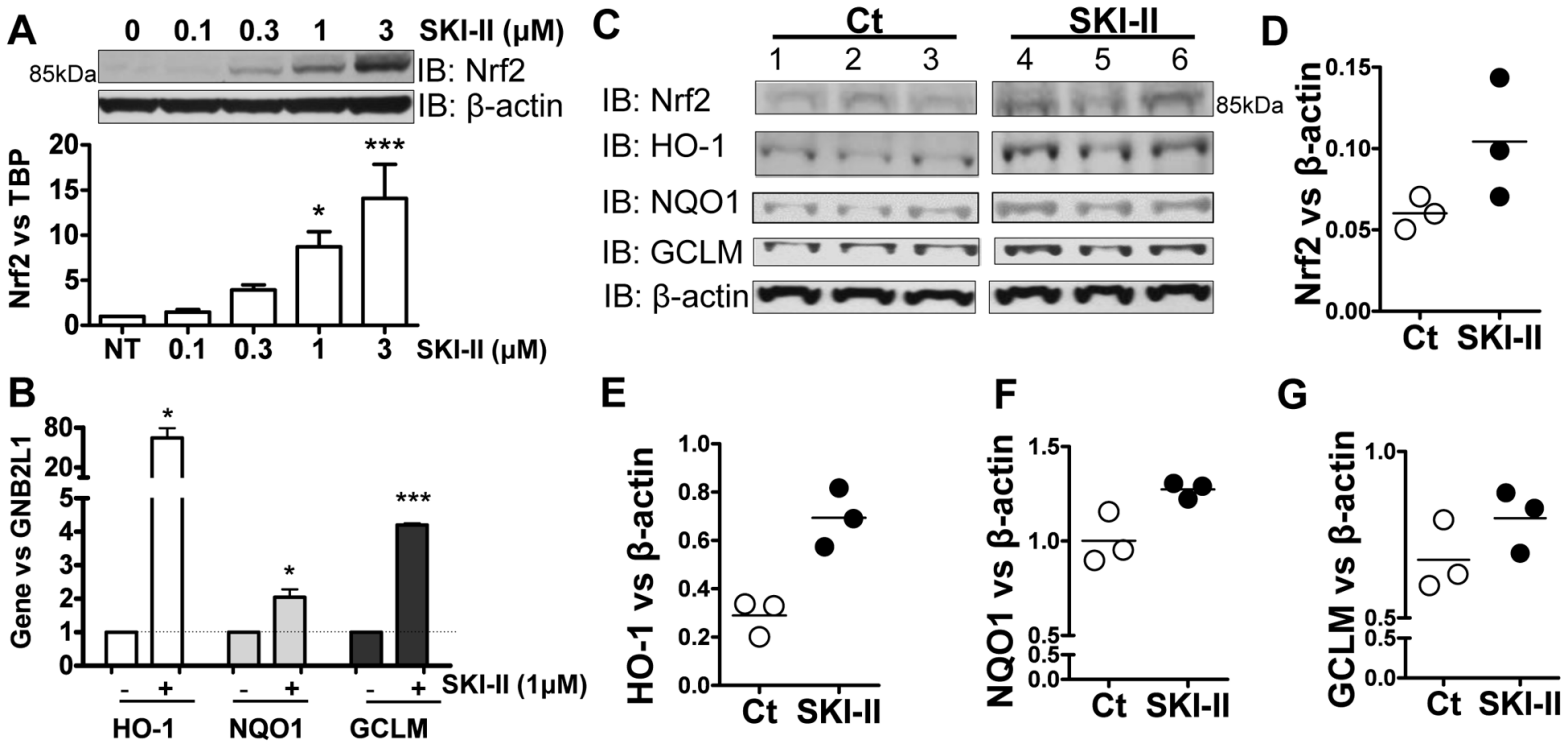
Effect of SKI-II on Nrf2 in primary human airway epithelial cells and in mice *in vivo*. **A.** Whole-cell extracts from normal human bronchial epithelial cells (HBEC) treated with increasing concentrations of SKI-II (0.1 to 3 µM) for 2 h were analysed by immunoblotting for Nrf2 and normalized using β-actin. ** p<0.001, * p<0.05. **B.** Gene expression of NQO1, GCLM and HO-1 were determined after 24 h treatment with SKI-II (1 µM) in HBEC. GNB2L1 was used as housekeeping gene. *** p<0.0001, * p<0.05. **C, D, E, F** and **G.** A/J mice were exposed to nebulized SKI-II (10 µM in PBS, n  =  3) or vehicle (Veh, n  =  3) for 2 h followed by a further 2 h exposure. Whole-cell extracts were analysed by immunoblotting for Nrf2, GCLM, HO-1 and NQO1 and normalized using β-actin expression. Results are representative of 3 independent experiments and are means and S.E. of triplicates.

**Figure 3:**
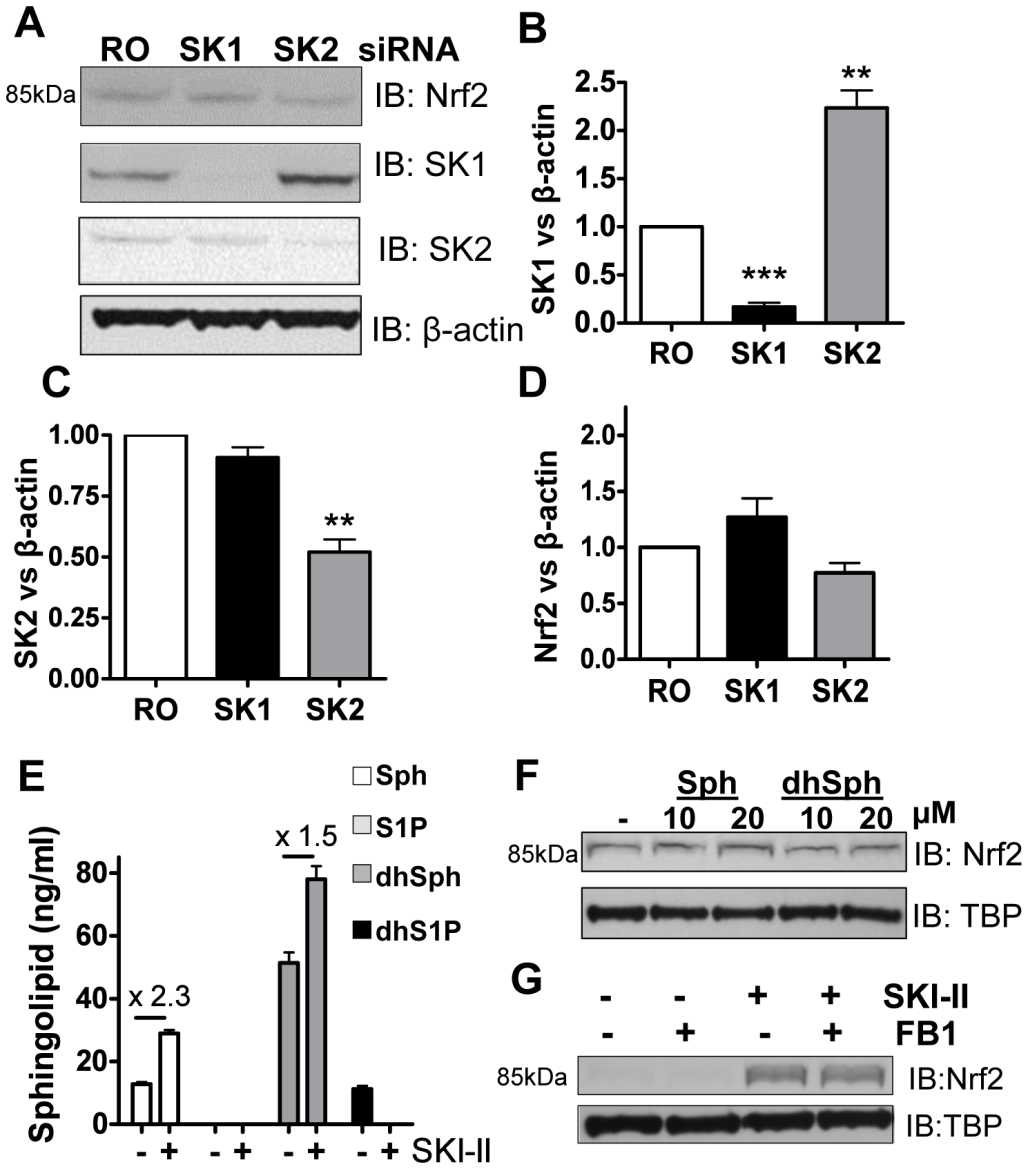
Effect of knocking-down SK1 and SK2 on Nrf2 expression in BEAS2B cells. A, B, C and D. Cells transfected with random oligonucleotide (RO) control, SK1 or SK2 siRNA were analysed by immunoblotting (IB) for Nrf2, SK1, SK2, and normalized using β-actin (fold change vs. NT). *** p<0.0001, ** p<0.001, * p<0.05. **E.** BEAS2B cells were stimulated with SKI-II (1 µM) for 2 h, pellets were spiked with C17 sphingosine, dihydrosphyngosine, S1P and dihydroS1P and extracted sphingolipids (C18) determined by LC-MS/MS. Intensity peaks for Sphingosine (Sph), dihydrosphingosine (dhSph) and dyhydrosphingosine 1 phosphate (dhS1P) are indicated in the graph whereas sphingosine 1 phophate levels (S1P) were below the detection range. **F.** Nuclear extracts from cells treated with either sphingosine (10 and 20 µM) or dihydrosphingosine (10 and 20 µM) for 2 h were analysed by immunoblotting for Nrf2 expression and normalized using TBP (fold change vs. NT). **G.** Nuclear extracts from BEAS2B cells treated with fumonisin B1 (FB1; 2 µM) for 1 hour and SKI-II (0.5 µM) for 2 h were analysed by immunoblotting for Nrf2 and TBP expression. Results are representative of two or more independent experiments and are means and S.E. of triplicates.

**Figure 4:**
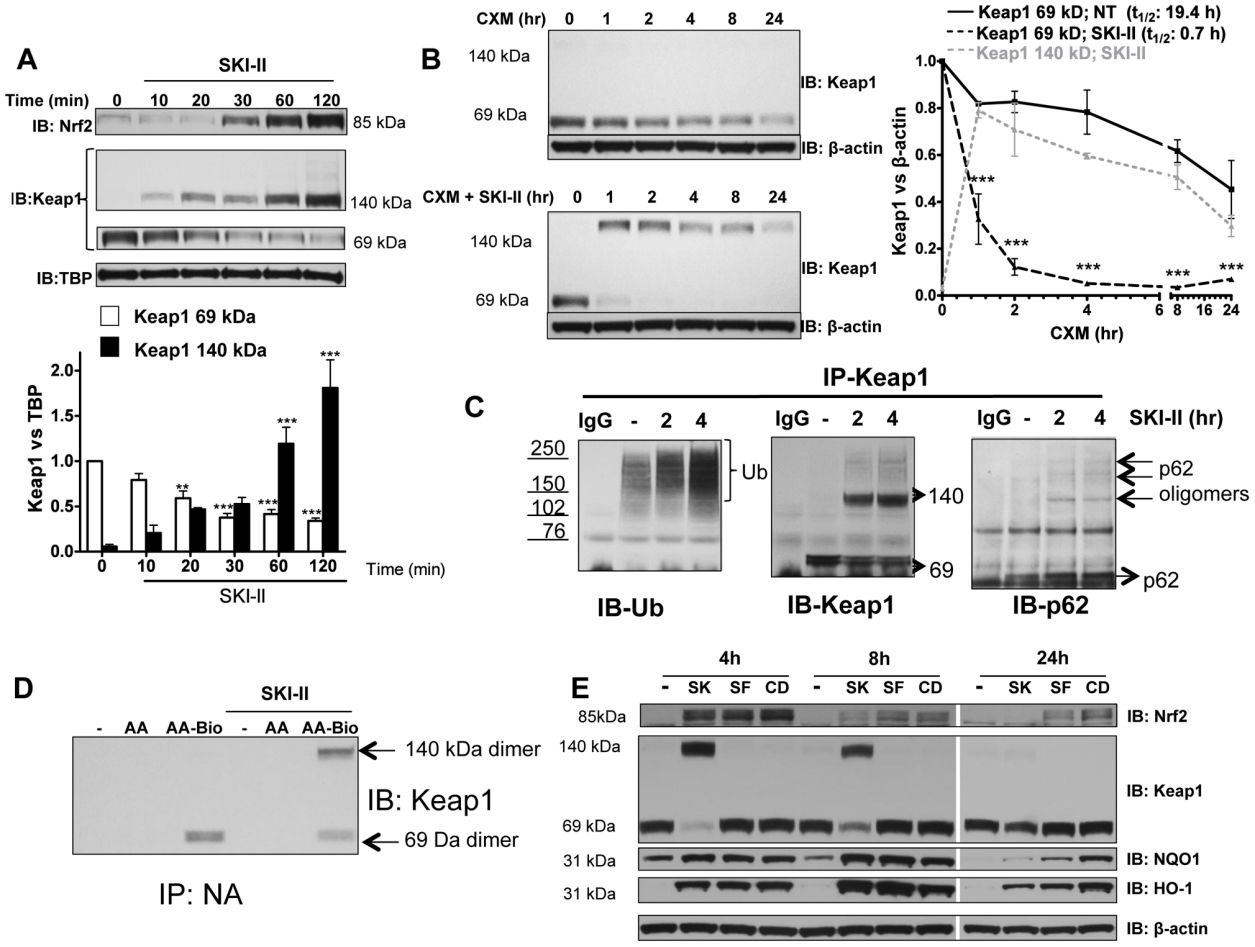
Effect of SKI-II on Keap1. **A.** Nuclear fractions from BEAS2B cells treated with SKI-II (1 µM) at increasing times (10–120 min) were analysed for Keap1 and Nrf2 expression and normalized using TBP (nuclear). Keap1 bands at 140 kDa and 69 kDa were analysed as fold change over non-treatment of the 69 kDa band. *** p<0.0001, ** p<0.001, * p<0.05. **B.** Cells were treated with cycloheximide (CXM) and SKI-II (1 µM) at different time points (1 to 24 h) and whole cell extracts were analysed for Keap1 expression normalized against β-actin. Keap1 bands at 140 kDa and 69 kDa were analysed as fold change over non-treatment of the 69 kDa band. *** p<0.0001 when CXM vs. CXM with SKI-II were compared. **C.** Whole-cell extracts from BEAS2B cells that were treated with SKI-II (1 µM) for 2 and 4 h were immunoprecipitated (IP) using a Keap1 antibody. IP Keap1 was analysed by immunoblotting for ubiquitin modification, p62 and Keap1 expression. IgG: Mouse immunoglobulin control (no cell lysates). Ub: ubiquitin. Hmw: High molecular weight. **D.** Whole-cell extracts from BEAS2B cells that were pre-treated with arachidonic acid (AA; 10 µM) or biotynilated arachidonic acid (AA-Bio; 10 µM) for 30 min were treated with SKI-II (1 µM) for 2.5 h and immunoprecipitated using Neutravidin Agarose Resin (IP-NA). IP-NA was analysed by immunoblotting for Keap1 expression. **E.** Whole-cell extracts from BEAS2B cells treated with SKI-II (SK; 1 µM), sulforaphane (SF; 5 µM) or CDDO-Imidazolide (CD; 50 nM) for 4 h, 8 h and 24 h were analysed by immunoblotting for Nrf2, Keap1, NQO1, HO-1 and β-actin. Results are representative of 3 independent experiments and are means and S.E. of triplicates.

**Figure 5:**
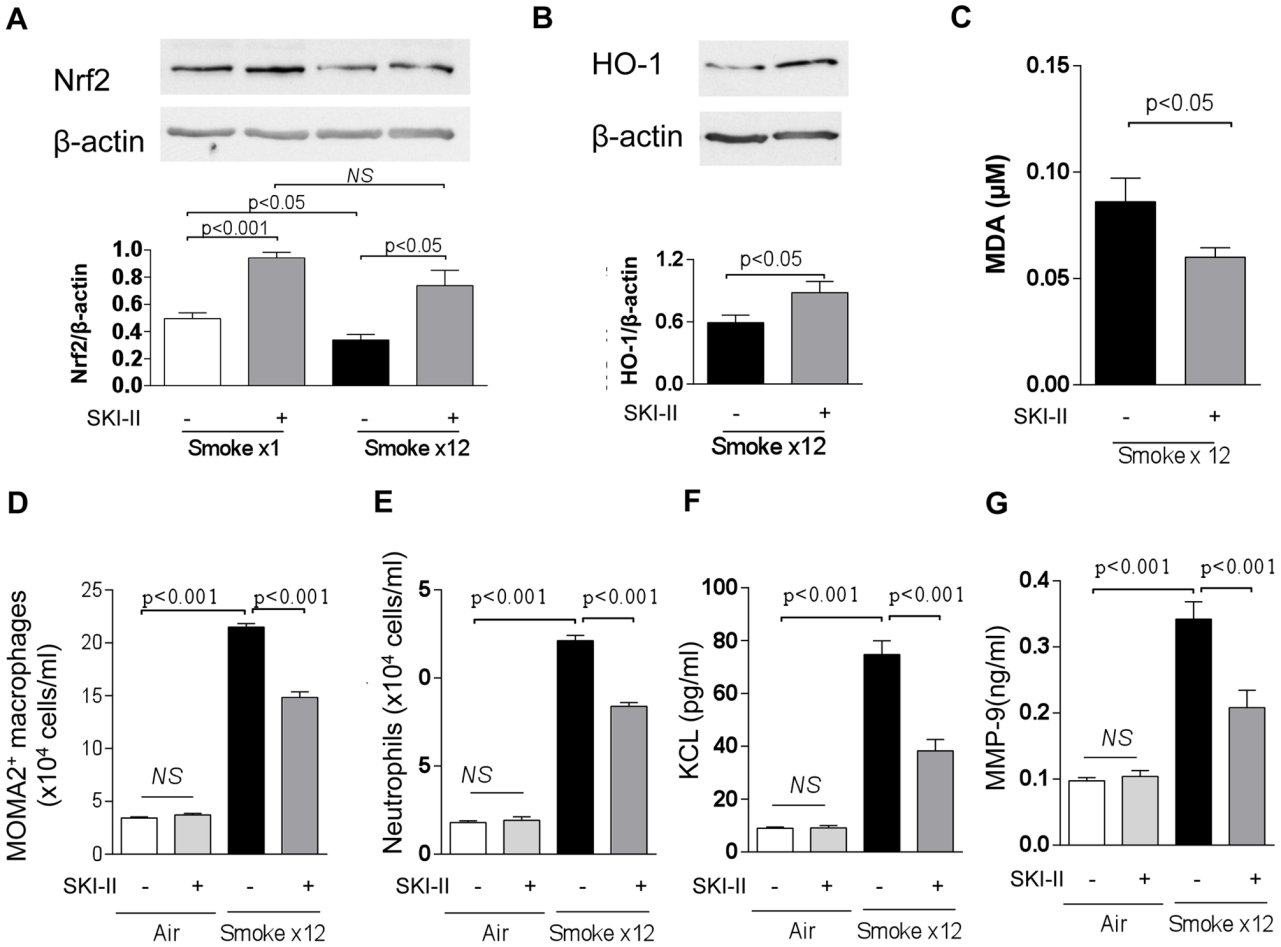
Effects of intranasal SKI-II on Nrf2, HO-1 and other inflammatory markers in cigarette smoke exposed mice. Nrf2 protein level normalized to β-actin in lung. Mice were exposed to smoke only once or 12 times (once daily). The lung was collected 2 h after cigarette smoke exposure. **B.** HO-1 protein in lung. The Western Blot image from pooled samples was shown. **C.** MDA in BALF, **D.** Alveolar macrophage in BALF, **E.** Neutrophils in BALF, **F.** CXCL1(KC) in BALF, **G.** MMP9 in BALF. Mice were treated with SKI-II (10 µM), intranasally before each cigarette smoke. Results are representative of 4–5 independent experiments and are means and S.E. of triplicates.

## Supporting Information

Figure S1 A. Effect of SK inhibition on Nrf2 in human airway epithelial cells (BEAS2B). Nuclear or whole cell extracts from cells treated with increasing concentrations of SK inhibitors DHS (0.3 to 30 µM), DMS (0.1 to 10 µM), SK1-I (0.1 to 10 µM) and FTY720 (0.1 to 10 µM) for 2 h were analysed by immunoblotting for Nrf2 expression and normalized using TBP or β-actin. **B.** BEAS2B cells were analysed for cell viability using an MTT assay 24 hours after SKI-II treatment. *** p<0.0001. **C.** BEAS2B cells were treated with cycloheximide (CXM) and SKI-II (1 µM) at different time points (1 to 24 h) and whole cell extracts were analysed for sphingosine kinase 1 (SK1) expression and β-actin. **D.** Cells transfected with random oligonucleotide (RO) control, SK1, SK2 and SK1+SK2 siRNA were analysed by immunoblotting (IB) for Nrf2, SK1, SK2 and β-actin. **E.** BEAS2B cells were stimulated with SKI-II (1 µM) for 2 h, pellets were spiked with C17 sphingosine, dihydrosphyngosine, S1P and dihydroS1P and extracted sphingolipids (C18) determined by LC-MS/MS. Intensity peaks for C17 and C18 sphingolipids are indicated in the graphs. Chromatograms show MRM traces as described in the Methods section. The higher levels observed for C18Sph and C18dhSph for the SKI-II treatment compared to NT can be seen from these chromatograms. **F.** Cells transfected with random oligonucleotide (RO) control, SK1+SK2 and Nrf2 siRNA were treated 24 h with SKI-II (0.1 to 10 µM) and analysed for cell viability using an MTT assay. Nrf2 KD was verified by immunoblotting (IB) for Nrf2 against β-actin. G. Cells transfected with random oligonucleotide (RO) control, SK1+SK2 and Nrf2 siRNA were treated 8 h with SKI-II (1 µM) and analysed for HO-1 expression by qRT-PCR.
(TIF)

Figure S2 A. Cytoplasmic fractions from BEAS2B treated with SKI-II (1 µM) at increasing times (10–120 min) were analysed for Keap1 and Nrf2 expression and normalized against β-actin (cytoplasmic). Keap1 bands at 140 kDa and 69 kDa were analysed as fold change over non-treatment of the 69 kDa band, *** p<0.0001, ** p<0.001, * p<0.05. **B.** Nuclear and cytoplasmic fractions from BEAS2B cells treated with SKI-II (0.5 µM) at increasing times (2–24 h) were analysed for the expressions of Keap1, Nrf2, TBP (nuclear) and β-actin (cytoplasmic). **C.** Whole cell extracts from BEAS2B cells were stimulated with SKI-II for 2 h and incubated in the presence of 10 µM 2′-7′-dichlorofluorescin diacetate (DCF-DA) for 30 min using H_2_O_2_ (50–500 µM) as control. **D.** Whole cell extracts (20 µg) from BEAS2B cells were stimulated SKI-II for 24 h used for the determination of the total anti-oxidant capacity by measuring the reduction of copper (II) to copper (I) against in µM copper reducing equivalents (CRE). Cells were also treated with cigarette smoke extract (3.5% v/v of 1 filtered cigarette into 10 ml of media) or N-acetyl cysteine (NAC, 10 mM) as controls 30 min before collection. **E.** BEAS2B cells were analysed for cell viability using an MTT assay 24 h after CSE treatment (3.5, 7.5 and 9% v/v) **F.** Whole-cell extracts from BEAS2B cells pre-treated with glutathione (GSH; 5 and 10 µM) before treatment with SKI-II (1 µM) for 2 h were analysed for Keap1 and Nrf2 expression and normalized against β-actin. **G.** BEAS2B cells were analysed for cell viability using an MTT assay 24 h after sulforpahane treatment (SF; 1 to 50 µM) or **H.** CDDO-Imidazolide (CDDO-Im; 10-250 nM) *** p<0.0001.
(TIF)
