# Supplementary material for: Activation of Transcription Factor Nrf2 Signalling by the Sphingosine Kinase Inhibitor SKI-II Is Mediated by the Formation of Keap1 Dimers
Source: PLoS One. 2014 Feb 5;9(2):e88168. doi: 10.1371/journal.pone.0088168 (PMC3914928; doi:10.1371/journal.pone.0088168)
Supplement: Figure S1 — A. Effect of SK inhibition on Nrf2 in human airway epithelial cells (BEAS2B). Nuclear or whole cell extracts from cells treated with increasing concentrations of SK inhibitors DHS (0.3 to 30 µM), DMS (0.1 to 10 µM), SK1-I (0.1 to 10 µM) and FTY720 (0.1 to 10 µM) for 2 h were analysed by immunoblotting for Nrf2 expression and normalized using TBP or β-actin. B. BEAS2B cells were analysed for cell viability using an MTT assay 24 hours after SKI-II treatment. *** p<0.0001. C. BEAS2B cells were treated with cycloheximide (CXM) and SKI-II (1 µM) at different time points (1 to 24 h) and whole cell extracts were analysed for sphingosine kinase 1 (SK1) expression and β-actin. D. Cells transfected with random oligonucleotide (RO) control, SK1, SK2 and SK1+SK2 siRNA were analysed by immunoblotting (IB) for Nrf2, SK1, SK2 and β-actin. E. BEAS2B cells were stimulated with SKI-II (1 µM) for 2 h, pellets were spiked with C17 sphingosine, dihydrosphyngosine, S1P and dihydroS1P and extracted sphingolipids (C18) determined by LC-MS/MS. Intensity peaks for C17 and C18 sphingolipids are indicated in the graphs. Chromatograms show MRM traces as described in the Methods section. The higher levels observed for C18Sph and C18dhSph for the SKI-II treatment compared to NT can be seen from these chromatograms. F. Cells transfected with random oligonucleotide (RO) control, SK1+SK2 and Nrf2 siRNA were treated 24 h with SKI-II (0.1 to 10 µM) and analysed for cell viability using an MTT assay. Nrf2 KD was verified by immunoblotting (IB) for Nrf2 against β-actin. G. Cells transfected with random oligonucleotide (RO) control, SK1+SK2 and Nrf2 siRNA were treated 8 h with SKI-II (1 µM) and analysed for HO-1 expression by qRT-PCR. (PPTX) [file pone.0088168.s001.pptx]

## Slide 1
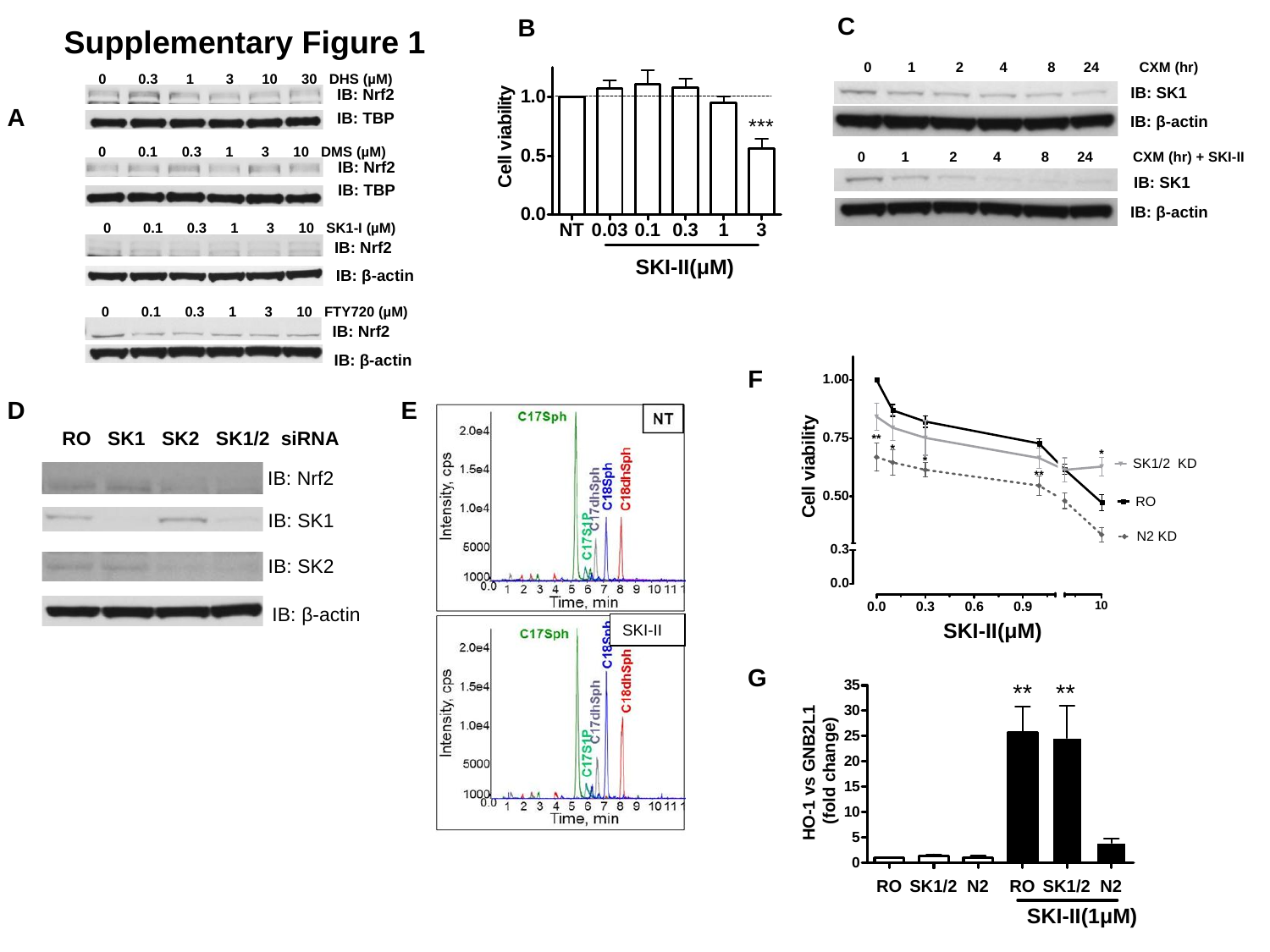

C
B
Supplementary Figure 1
0 1 2 4 8 24 CXM (hr)
0 0.3 1 3 10 30 DHS (µM)
IB: SK1
IB: Nrf2
A
IB: TBP
IB: β-actin
0 0.1 0.3 1 3 10 DMS (µM)
0 1 2 4 8 24 CXM (hr) + SKI-II
IB: Nrf2
IB: SK1
IB: TBP
IB: β-actin
0 0.1 0.3 1 3 10 SK1-I (µM)
IB: Nrf2
SKI-II(μM)
IB: β-actin
0 0.1 0.3 1 3 10 FTY720 (µM)
IB: Nrf2
IB: β-actin
F
E
D
SKI-II
RO SK1 SK2 SK1/2 siRNA
IB: Nrf2
IB: SK1
IB: SK2
IB: β-actin
SKI-II(μM)
G
SKI-II(1μM)
