# Supplementary material for: Activation of Transcription Factor Nrf2 Signalling by the Sphingosine Kinase Inhibitor SKI-II Is Mediated by the Formation of Keap1 Dimers
Source: PLoS One. 2014 Feb 5;9(2):e88168. doi: 10.1371/journal.pone.0088168 (PMC3914928; doi:10.1371/journal.pone.0088168)
Supplement: Figure S2 — A. Cytoplasmic fractions from BEAS2B treated with SKI-II (1 µM) at increasing times (10–120 min) were analysed for Keap1 and Nrf2 expression and normalized against β-actin (cytoplasmic). Keap1 bands at 140 kDa and 69 kDa were analysed as fold change over non-treatment of the 69 kDa band, *** p<0.0001, ** p<0.001, * p<0.05. B. Nuclear and cytoplasmic fractions from BEAS2B cells treated with SKI-II (0.5 µM) at increasing times (2–24 h) were analysed for the expressions of Keap1, Nrf2, TBP (nuclear) and β-actin (cytoplasmic). C. Whole cell extracts from BEAS2B cells were stimulated with SKI-II for 2 h and incubated in the presence of 10 µM 2′-7′-dichlorofluorescin diacetate (DCF-DA) for 30 min using H2O2 (50–500 µM) as control. D. Whole cell extracts (20 µg) from BEAS2B cells were stimulated SKI-II for 24 h used for the determination of the total anti-oxidant capacity by measuring the reduction of copper (II) to copper (I) against in µM copper reducing equivalents (CRE). Cells were also treated with cigarette smoke extract (3.5% v/v of 1 filtered cigarette into 10 ml of media) or N-acetyl cysteine (NAC, 10 mM) as controls 30 min before collection. E. BEAS2B cells were analysed for cell viability using an MTT assay 24 h after CSE treatment (3.5, 7.5 and 9% v/v) F. Whole-cell extracts from BEAS2B cells pre-treated with glutathione (GSH; 5 and 10 µM) before treatment with SKI-II (1 µM) for 2 h were analysed for Keap1 and Nrf2 expression and normalized against β-actin. G. BEAS2B cells were analysed for cell viability using an MTT assay 24 h after sulforpahane treatment (SF; 1 to 50 µM) or H.CDDO-Imidazolide (CDDO-Im; 10-250 nM) *** p<0.0001. (PPTX) [file pone.0088168.s002.pptx]

## Slide 1
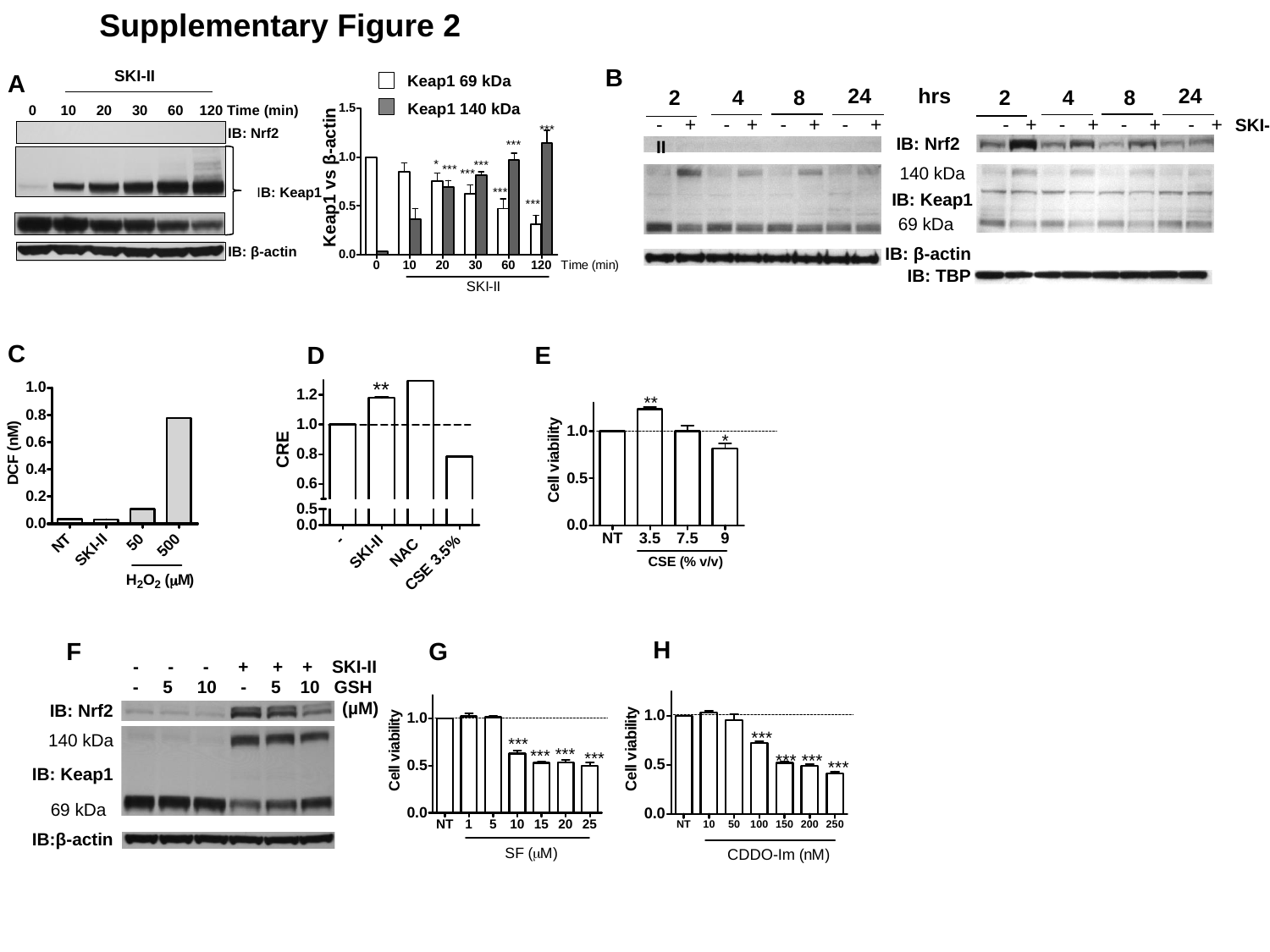

Supplementary Figure 2
B
SKI-II
A
Keap1 69 kDa
hrs
24
24
4
2
4
8
2
8
Keap1 140 kDa
0 10 20 30 60 120 Time (min)
- + - + - + - + - + - + - + - + SKI-II
IB: Nrf2
IB: Nrf2
140 kDa
Keap1 vs β-actin
IB: Keap1
IB: Keap1
69 kDa
IB: β-actin
IB: β-actin
IB: TBP
C
E
D
H
F
G
 - - - + + + SKI-II
 - 5 10 - 5 10 GSH
 (µM)
IB: Nrf2
140 kDa
IB: Keap1
69 kDa
IB:β-actin
