# Supplementary figures and images for: Correction: Activation of Transcription Factor Nrf2 Signalling by the Sphingosine Kinase Inhibitor SKI-II Is Mediated by the Formation of Keap1 Dimers
Source: PLoS One. 2014 May 2;9(5):e97208. doi: 10.1371/journal.pone.0097208 (PMC4008611; doi:10.1371/journal.pone.0097208)

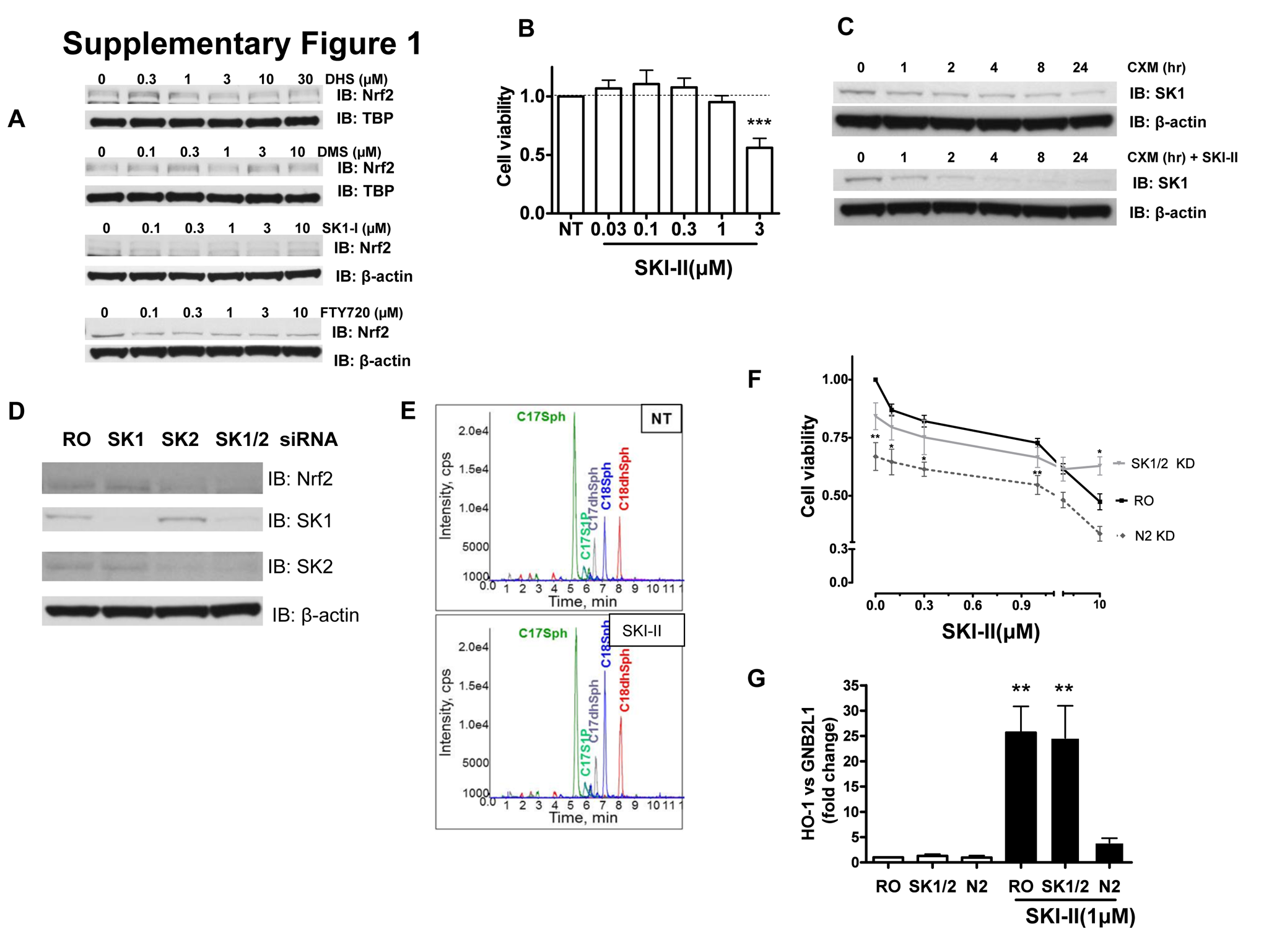

Supplement: Figure S1 — A. Effect of SK inhibition on Nrf2 in human airway epithelial cells (BEAS2B). Nuclear or whole cell extracts from cells treated with increasing concentrations of SK inhibitors DHS (0.3 to 30 µM), DMS (0.1 to 10 µM), SK1-I (0.1 to 10 µM) and FTY720 (0.1 to 10 µM) for 2 h were analysed by immunoblotting for Nrf2 expression and normalized using TBP or β-actin. B. BEAS2B cells were analysed for cell viability using an MTT assay 24 hours after SKI-II treatment. *** p<0.0001. C. BEAS2B cells were treated with cycloheximide (CXM) and SKI-II (1 µM) at different time points (1 to 24 h) and whole cell extracts were analysed for sphingosine kinase 1 (SK1) expression and β-actin. D. Cells transfected with random oligonucleotide (RO) control, SK1, SK2 and SK1+SK2 siRNA were analysed by immunoblotting (IB) for Nrf2, SK1, SK2 and β-actin. E. BEAS2B cells were stimulated with SKI-II (1 µM) for 2 h, pellets were spiked with C17 sphingosine, dihydrosphyngosine, S1P and dihydroS1P and extracted sphingolipids (C18) determined by LC-MS/MS. Intensity peaks for C17 and C18 sphingolipids are indicated in the graphs. Chromatograms show MRM traces as described in the Methods section. The higher levels observed for C18Sph and C18dhSph for the SKI-II treatment compared to NT can be seen from these chromatograms. F. Cells transfected with random oligonucleotide (RO) control, SK1+SK2 and Nrf2 siRNA were treated 24 h with SKI-II (0.1 to 10 µM) and analysed for cell viability using an MTT assay. Nrf2 KD was verified by immunoblotting (IB) for Nrf2 against β-actin. G. Cells transfected with random oligonucleotide (RO) control, SK1+SK2 and Nrf2 siRNA were treated 8 h with SKI-II (1 µM) and analysed for HO-1 expression by qRT-PCR. (TIF) [file pone.0097208.s001.tif]

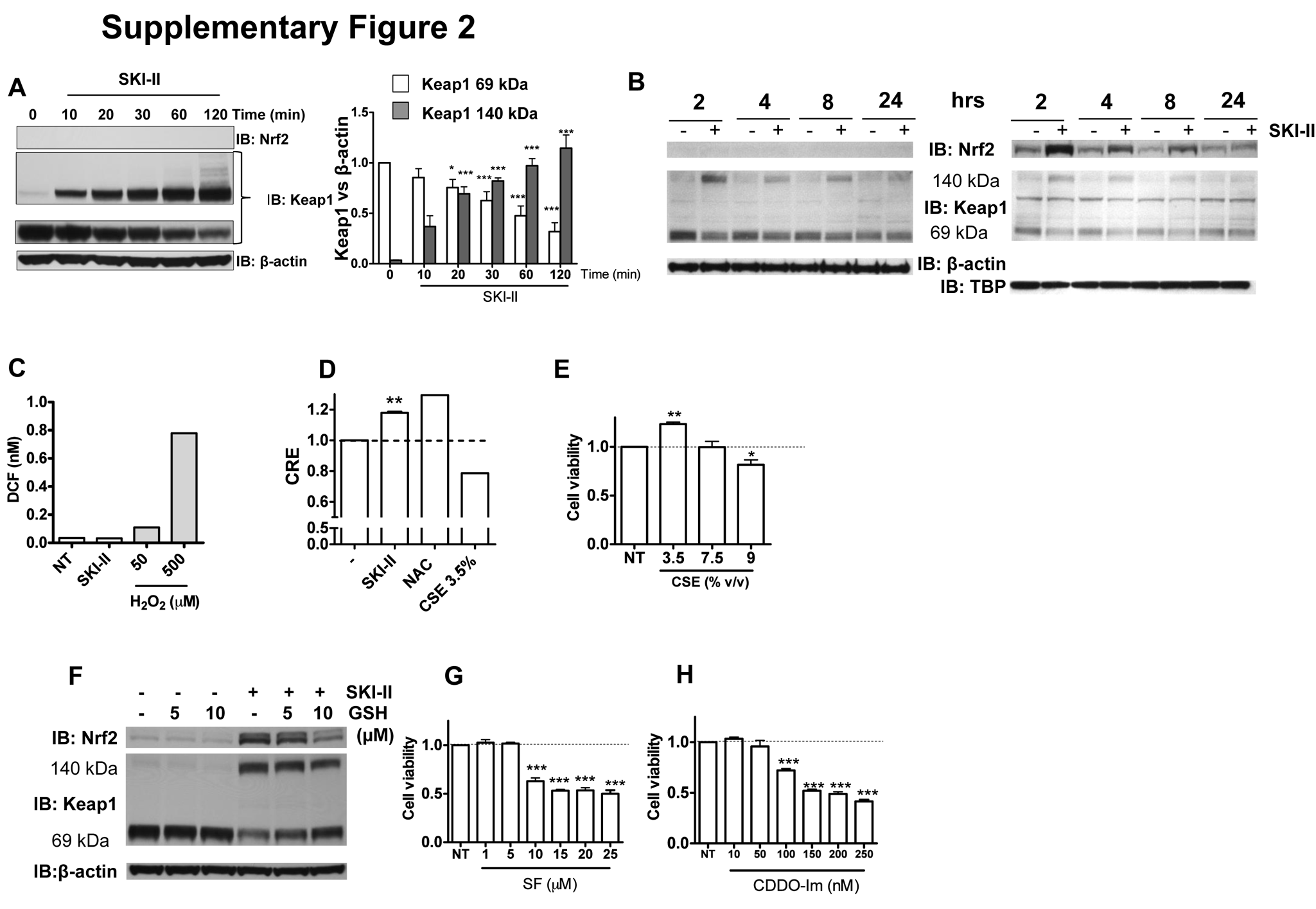

Supplement: Figure S2 — A. Cytoplasmic fractions from BEAS2B treated with SKI-II (1 µM) at increasing times (10–120 min) were analysed for Keap1 and Nrf2 expression and normalized against β-actin (cytoplasmic). Keap1 bands at 140 kDa and 69 kDa were analysed as fold change over non-treatment of the 69 kDa band, *** p<0.0001, ** p<0.001, * p<0.05. B. Nuclear and cytoplasmic fractions from BEAS2B cells treated with SKI-II (0.5 µM) at increasing times (2–24 h) were analysed for the expressions of Keap1, Nrf2, TBP (nuclear) and β-actin (cytoplasmic). C. Whole cell extracts from BEAS2B cells were stimulated with SKI-II for 2 h and incubated in the presence of 10 µM 2′-7′-dichlorofluorescin diacetate (DCF-DA) for 30 min using H2O2 (50–500 µM) as control. D. Whole cell extracts (20 µg) from BEAS2B cells were stimulated SKI-II for 24 h used for the determination of the total anti-oxidant capacity by measuring the reduction of copper (II) to copper (I) against in µM copper reducing equivalents (CRE). Cells were also treated with cigarette smoke extract (3.5% v/v of 1 filtered cigarette into 10 ml of media) or N-acetyl cysteine (NAC, 10 mM) as controls 30 min before collection. E. BEAS2B cells were analysed for cell viability using an MTT assay 24 h after CSE treatment (3.5, 7.5 and 9% v/v) F. Whole-cell extracts from BEAS2B cells pre-treated with glutathione (GSH; 5 and 10 µM) before treatment with SKI-II (1 µM) for 2 h were analysed for Keap1 and Nrf2 expression and normalized against β-actin. G. BEAS2B cells were analysed for cell viability using an MTT assay 24 h after sulforpahane treatment (SF; 1 to 50 µM) or H. CDDO-Imidazolide (CDDO-Im; 10-250 nM) *** p<0.0001. (TIF) [file pone.0097208.s002.tif]
